# Genome-Wide Epigenetic Regulation of Gene Transcription in Maize Seeds

**DOI:** 10.1371/journal.pone.0139582

**Published:** 2015-10-15

**Authors:** Xiaoduo Lu, Weixuan Wang, Wen Ren, Zhenguang Chai, Wenzhu Guo, Rumei Chen, Lei Wang, Jun Zhao, Zhihong Lang, Yunliu Fan, Jiuran Zhao, Chunyi Zhang

**Affiliations:** 1 School of Life Sciences, Qilu Normal University, Jinan, 250200, China; 2 Department of Crop Genomics & Genetic Improvement, Biotechnology Research Institute, Chinese Academy of Agricultural Sciences, Beijing, 100081, China; 3 National Key Facility for Crop Gene Resources and Genetic Improvement (NFCRI), Beijing, 100081, People’s Republic of China; 4 Maize Research Center, Beijing Academy of Agriculture and Forestry Sciences, Beijing, 100097, China; National Taiwan University, TAIWAN

## Abstract

**Background:**

Epigenetic regulation is well recognized for its importance in gene expression in organisms. DNA methylation, an important epigenetic mark, has received enormous attention in recent years as it’s a key player in many biological processes. It remains unclear how DNA methylation contributes to gene transcription regulation in maize seeds. Here, we take advantage of recent technologies to examine the genome-wide association of DNA methylation with transcription of four types of DNA sequences, including protein-coding genes, pseudogenes, transposable elements, and repeats in maize embryo and endosperm, respectively.

**Results:**

The methylation in CG, CHG and CHH contexts plays different roles in the control of gene expression. Methylation around the transcription start sites and transcription stop regions of protein-coding genes is negatively correlated, but in gene bodies positively correlated, to gene expression level. The upstream regions of protein-coding genes are enriched with 24-nt siRNAs and contain high levels of CHH methylation, which is correlated to gene expression level. The analysis of sequence content within CG, CHG, or CHH contexts reveals that only CHH methylation is affected by its local sequences, which is different from Arabidopsis.

**Conclusions:**

In summary, we conclude that methylation-regulated transcription varies with the types of DNA sequences, sequence contexts or parts of a specific gene in maize seeds and differs from that in other plant species. Our study helps people better understand from a genome-wide viewpoint that how transcriptional expression is controlled by DNA methylation, one of the important factors influencing transcription, and how the methylation is associated with small RNAs.

## Introduction

Cytosine methylation, an epigenetic marker, is important for transposable element (TE) silencing, gene expression and gene imprinting in vertebrates, flowering plants, and some fungi. Global demethylation of genomic DNA strongly reactivates TE transcription in mammals and plants [1–4]. Decreased DNA methylation in *Arabidopsis thaliana* leads to retrotransposon mobilization and TE activation and results in the increase of TE copy number [4]. In mammals, DNA methylation patterns are established and maintained by DNA methyltransferase 3 (DNMT3) and methyltransferase DNMT1, respectively [1, 5, 6]. In plants, DOMAINS REARRANGED METHYLTRANSFERASE2 (DRM2), the plant homologue of DNMT3, catalyzes *de novo* methylation; MET1, the plant homologue of DNMT1, maintains CG methylation. CHG methylation is maintained by CHROMOMETHYLASE 3 (CMT3), a plant-specific DNA methyltransferase. *de novo* methylation mechanism by DRM2 is responsible for the maintenance of CHH methylation [7].

Endogenous small interfering RNAs (siRNAs) are the best characterized small RNAs that defend eukaryotic cells against TE mobilization in plants. siRNAs regulate TE activity primarily through RNA-directed DNA methylation (RdDM) [8]. Two plant-specific RNA polymerases, Pol IV and Pol V, are involved in RdDM. Pol IV initiates 24-nucleotide (nt) siRNA biogenesis by transcribing long single-stranded RNAs (ssRNAs). RNA-dependent RNA polymerase 2 (RDR2) utilizes the ssRNAs as templates to generate double-stranded RNAs (dsRNAs) which are processed into 24-nt siRNAs by DICER-like 3 (DCL3). 24-nt siRNAs are loaded into AGO4 which interacts with NUCLEAR RNA POLYMERASE E1 (NRPE1), a Pol V subunit [8–11]. Pol V functions to produce intergenic noncoding (IGN) transcripts which are essential for DNA methylation and silencing of surrounding loci, but not to produce 24-nt siRNAs [12]. A complex comprising the AGO4-siRNAs and a number of other proteins (including DRM2) triggers local DNA methylation [13–15].

Maize seeds are not only one of the most important crop materials which provide resource for food, feed, biofuel and raw material for processing, but also an important model organism for fundamental research of genetics and genomics [16]. Epigenetic regulation of gene expression is crucial for seed development [17]. Recently, we reported that the epigenetic machinery is probably operating in the early developing maize seed [18]. To advance our understanding of epigenetic networking in maize seed, highly integrated epigenome maps for 9-DAP (days after pollination) embryo and endosperm of maize B73 are constructed via deep sequencing of the cytosine methylome (methylC-seq), transcriptome (mRNA-seq), and small RNA transcriptome (sRNA-seq). The dataset will aid to understand the epigenetic mechanisms underlying gene expression in the early developing maize seeds.

## Results

### Bisulfite sequencing of the maize seed genome

**Fig 1:**
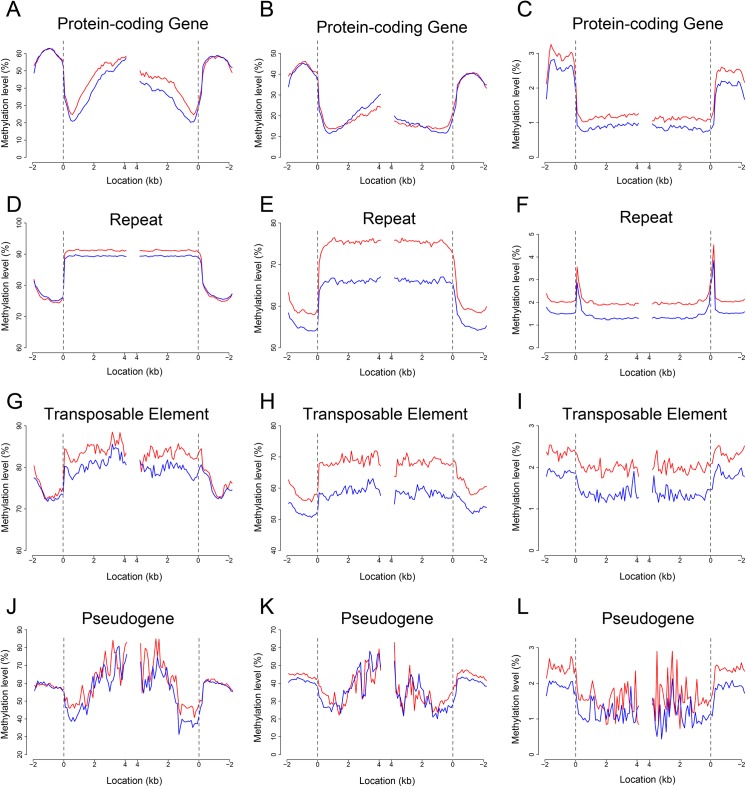
DNA methylation profiles in the embryo and endosperm. (A to L) Maize B73 RefGen_V2-annotated protein-coding genes [(A), (B), and (C)], repeat regions [(D), (E), and (F)], transposable elements [(G), (H), and (I)] and pseudogenes [(J), (K), and (L)] are aligned at the 5’ end (left) or the 3’end (right), and average methylation levels for each 50-nt interval are plotted from 2 kb away from the gene (negative numbers) to 4 kb into the gene (positive numbers). Embryo methylation is represented by the red trace and endosperm by the blue trace. The dashed line at zero represents the point of alignment. CG methylation is shown in (A), (D), (G), (J), CHG in (B), (E), (H), (K), and CHH in (C), (F), (I) and (L).

To decipher DNA methylation landscapes at early stage of maize seeds, we isolated genomic DNA from 9-DAP embryo and endosperm of maize inbred line B73, and performed MethylC-seq to identify cytosines that are methylated. The embryos were characterized with emerging primordia and the endosperm just completed differentiation, with aleurone and transfer cell as well as starchy endosperm cells formed [18], indicating an important developmental stage of the seeds. MethylC sequencing yielded 433,715,164 and 456,749,505 reads for the embryo and endosperm, respectively (Table A in S1 File). Among those, 165 million reads (38.11%, embryo) and 191 million reads (41.93%, endosperm) were aligned to unique locations of the B73 reference genome. The cytosines (2,936,910,521 from the embryo and 3,523,921,294 from the endosperm) were aligned to unique positions and covered 33.65% and 35.64% of the total genomic cytosines with average read depths of 9- and 10-fold coverage of each DNA strand, respectively (Table B in S1 File). Like other flowering plants, cytosine methylation occurred in CG, CHG (H is A, C or T) and CHH sequence contexts in both embryo and endosperm of maize. The bulk cytosine methylation frequency was 80.26% for CG, 63.81% for CHG, and 2.51% for CHH in embryo, and 78.40% for CG, 57.60% for CHG and 1.82% for CHH in endosperm (Table A in S1 File), indicating the maize endosperm genome was hypo-methylated compared to the embryo genome (Fig 1; Table C in S1 File). 87% of the CG contexts were methylated, out of which more than 70% were heavily methylated (80%–100%). Similar to CG, over 80% of CHG was methylated in both the embryo and endosperm, the majority of which were heavily methylated (80–100%), while CHH was markedly less methylated compared to CG (Fig A and Table B in S1 File).

### Methylation profiles of 9-DAP maize embryo and endosperm

Overall, the maize endosperm genome was hypomethylated compared to the embryo genome (Fig 1; Table C in S1 File), which is in agreement with previous reports [19, 20]. Higher CG methylation in the embryo compared to endosperm was found mainly in the transcribed regions of protein-coding genes and TEs as well as in repeat regions (Fig 1A, 1D and 1G). However, CHG methylation was slightly higher in the endosperm than the embryo in the middle part of the transcribed region of protein-coding genes (Fig 1B), and significantly higher in the embryo than the endosperm in upstream to downstream repeat regions and TEs (Fig 1E and 1H). CHH methylation was consistently higher in the embryo than the endosperm (Fig 1C, 1F, 1I and 1L). There was no significant difference at CG context between embryo and endosperm, while the methylation level at CHG and CHH context is lower in endosperm than embryo. This pattern is similar to rice [21], another monocotyledon plant, and different from Arabidopsis [20], a dicotyledon plant. 87% methylated CGs were observed, among which more than 70% were highly methylated (80%–100%). Unlike CGs, CHHs were either demethylated or hypomethylated both in embryo and endosperm (Fig A in S1 File).

To further identify sequences that are differentially methylated in the embryo compared with the endosperm, differential methylation regions (DMRs) were determined. We calculated fractional methylation in each context within 50-base pair (bp) windows and subtracted endosperm methylation from embryo methylation. The results showed that DNA methylation differences between the embryo and endosperm varied at genomic loci subsets (Fig B and Table C in S1 File). 421,137 and 415,490 discreet DMRs corresponding to 24,341,600 and 24,041,950 bp in CG methylation were identified in sense and antisense strand, respectively. 285,017 (67.68%) and 281,796 (67.82%) of those DMRs were highly methylated in embryo in sense and antisense strand, respectively (Table D in S1 File). In CHG context, 738,334 (47,402,500 bp) and 736,262 (47,335,050 bp) loci were more methylated in sense and antisense strand, respectively. About 78% (580,449 loci in sense strand and 578,949 loci in antisense strand) of these DMRs were more methylated in embryo than in endosperm. We also found 577,714 (31,658,700 bp) and 577,009 (31,664,700) loci with change in CHH methylation in sense and antisense strand, respectively. 63.1% (364,486 loci in sense strand and 364,239 loci in antisense strand) of the loci were highly methylated in embryo in comparison to endosperm (Table D in S1 File). Notably, around 22% and 37% of identified loci were hypermethylated at CHG and CHH, respectively, in endosperm. Surprisingly, about one third of the loci identified were hypermethylated at CG in endosperm, which is much higher than that in Arabidopsis [20].

Higher CG methylation in the embryo compared to endosperm was found mainly in the transcribed regions of protein-coding genes and TEs as well as in repeat regions (Fig 1A, 1D and 1G). However, CHG methylation was slightly higher in the endosperm than the embryo in the middle part of the transcribed region of protein-coding genes (Fig 1B), and significantly higher in the embryo than the endosperm in upstream to downstream repeat regions and TEs (Fig 1E and 1H). CHH methylation was consistently higher in the embryo than the endosperm (Fig 1C, 1F, 1I and 1L).

CG, CHG and CHH methylation were lowest from 600 bp to 700 bp downstream of the transcription start site (TSS) within the transcript, and a similar pattern was also observed at the 3’ end of genes (Fig 1A, 1B and 1C), which differs from rice, *Arabidopsis*, and human [21–24]. CG and CHG methylation patterns were somewhat similar between repeats and TEs (Fig 1D, 1E, 1G and 1H), while CHH methylation differed significantly (Fig 1F and 1I). Interestingly, the CG and CHG methylation patterns in the transcribed regions of pseudogenes were similar to those of protein-coding genes, but the methylation level of pseudogenes was significantly higher than that of protein-coding genes (40–80% in pseudogenes *vs*. 20–60% in protein-coding genes for CG; 20–60% in pseudogenes *vs*. 10–30% in protein-coding genes for CHG; Fig 1A, 1B, 1J and 1K), suggesting a correlation between enhanced methylation and pseudogene inactivation.

We observed that CHH methylation pattern differed from CG or CHG. Both CG and CHG were increasingly methylated from the 5’ end inwards and decreasingly methylated towards the 3’ end in protein-coding genes and pseudogenes (Fig 1A, 1B, 1J and 1K); CG and CHG were evenly methylated in repeat regions (Fig 1D and 1E), but less evenly methylated in transcribed regions of TEs (Fig 1G and 1H). In contrast, CHG methylation was almost absent in transcribed regions in Arabidopsis and rice [21, 23]. Unlike CG or CHG, CHH was methylated at the lowest frequencies in the transcribed regions of protein-coding genes and TEs as well as in repeat regions compared to other regions of the genes (Fig 1C, 1F and 1I), peaking at the two ends of repeats (Fig 1F).

### Local sequence effects on DNA methylation

**Fig 2:**
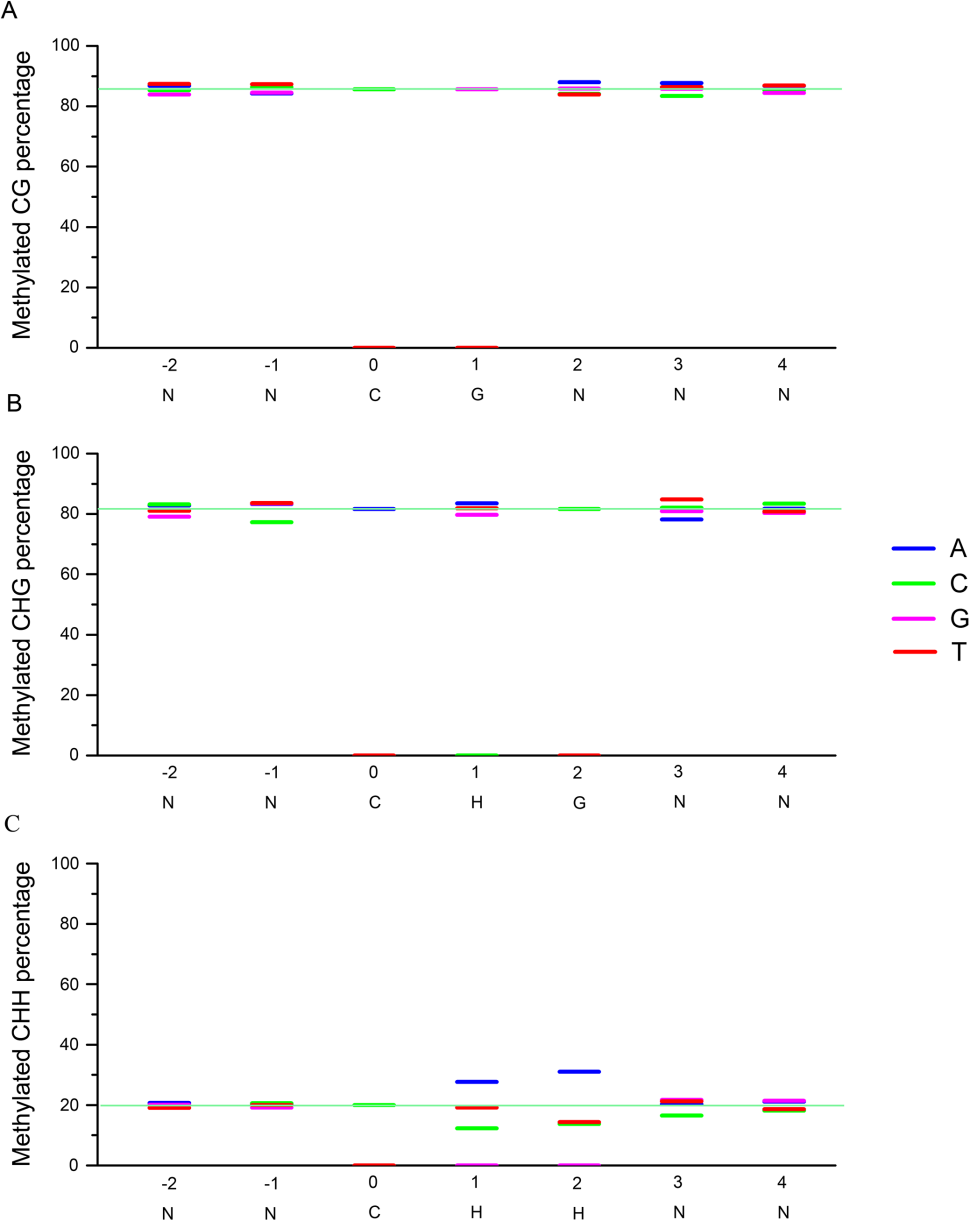
Local sequence effects on DNA methylation in the embryo. Sequence contexts that are preferentially methylated in the embryo for 7-mer sequences, in which the methylated cytosine is in the third position. (A), CG context in the embryo; (B), CHG context in the embryo; (C), CHH context in the embryo. The y axis indicates the methylation level and the x axis indicates the base composition and position.

To explore the local sequence effects on DNA methylation, the upstream two nucleotides and downstream four nucleotides surrounding cytosines were assessed in terms of their effects on cytosine methylation (Fig 2; S1 Table). Strong effects were found in a CHH context. A cytosine immediately followed by another cytosine was less likely to be methylated than a cytosine neighboring a thymidine or adenine; in contrast, a cytosine immediately followed by an adenine was more likely to be methylated (Fig 2C). This was clearly demonstrated by the observation that CAH sites were methylated at a level twofold higher than CCH sites in both the embryo and the endosperm (Fig 2C). As opposed to the slightly repressive effect of cytosines at positions + 1, + 2 or + 3, adenosines at the 3’ end of the CHH context were associated with an increase in cytosine methylation frequency. This effect was strongest at the + 2 positions where a CHA was methylated twofold more frequently than CHC or CHT (Fig 2). The sequence effect in the CHH context on DNA methylation was also observed in the endosperm (Fig C in S1 File; S1 Table), and was conserved between maize and *Arabidopsis*. However, only minor effects were observed for CHG or CG context, which is different from Arabidopsis [25].

### The association of small RNAs with DNA methylation

**Fig 3:**
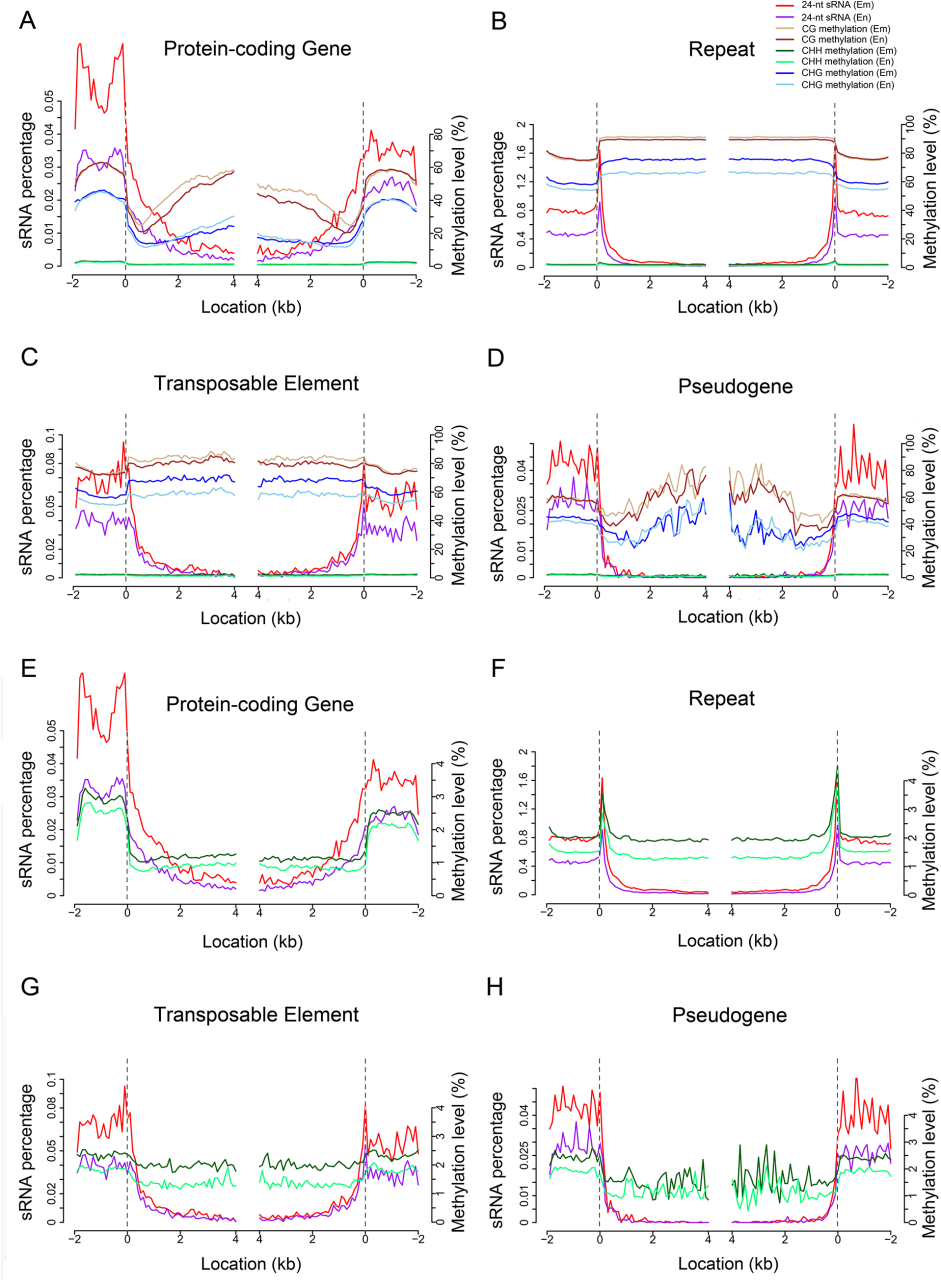
Correlation between 24-nt sRNA and DNA methylation in the embryo and endosperm. (A to D) Correlations between 24-nt sRNA and DNA methylation in protein-coding genes (A), repeats (B), transposable elements (C) and pseudogenes (D). (E to H) Correlations between 24-nt sRNA and CHH methylation in protein-coding genes (A), repeats (B), transposable elements (C) and pseudogenes (D). The dashed line at zero represents the point of alignment.

Previously it was demonstrated that a subset of small RNAs (sRNAs) pool targets DNA methylation through RdDM [26], an essential process for the establishment of DNA methylation and its maintenance in asymmetric contexts. To characterize the relationship between sRNAs and genome methylation in maize seed, we first performed deep sequencing of sRNAs from the embryo and endosperm, respectively, and then investigated the correlation between sRNA production and DNA methylation. We found that 24-nt sRNAs were significantly more abundant in the upstream and downstream regions of genes in the embryo than in the endosperm (Fig 3); in contrast, 21-, 22- or 23-nt sRNAs were produced at higher levels in the endosperm than in the embryo (Fig D-F in S1 File). A significant positive correlation between CHH methylation and 24-nt sRNA accumulation was found mainly in the upstream region of protein-coding genes and pseudogenes (Fig 3E and 3H) and in the two ends of repeats (Fig 3F), but we did not observe any correlation between CG/CHG methylation and 24-nt sRNA production (Fig 3A–3D). Similar relationships were also observed for 21-, 22-, or 23-nt sRNAs (Fig D-I in S1 File), suggesting that the functions of those sRNAs may differ from those of 24-nt sRNAs.

### siRNA-regulated gene expression in maize seeds

**Fig 4:**
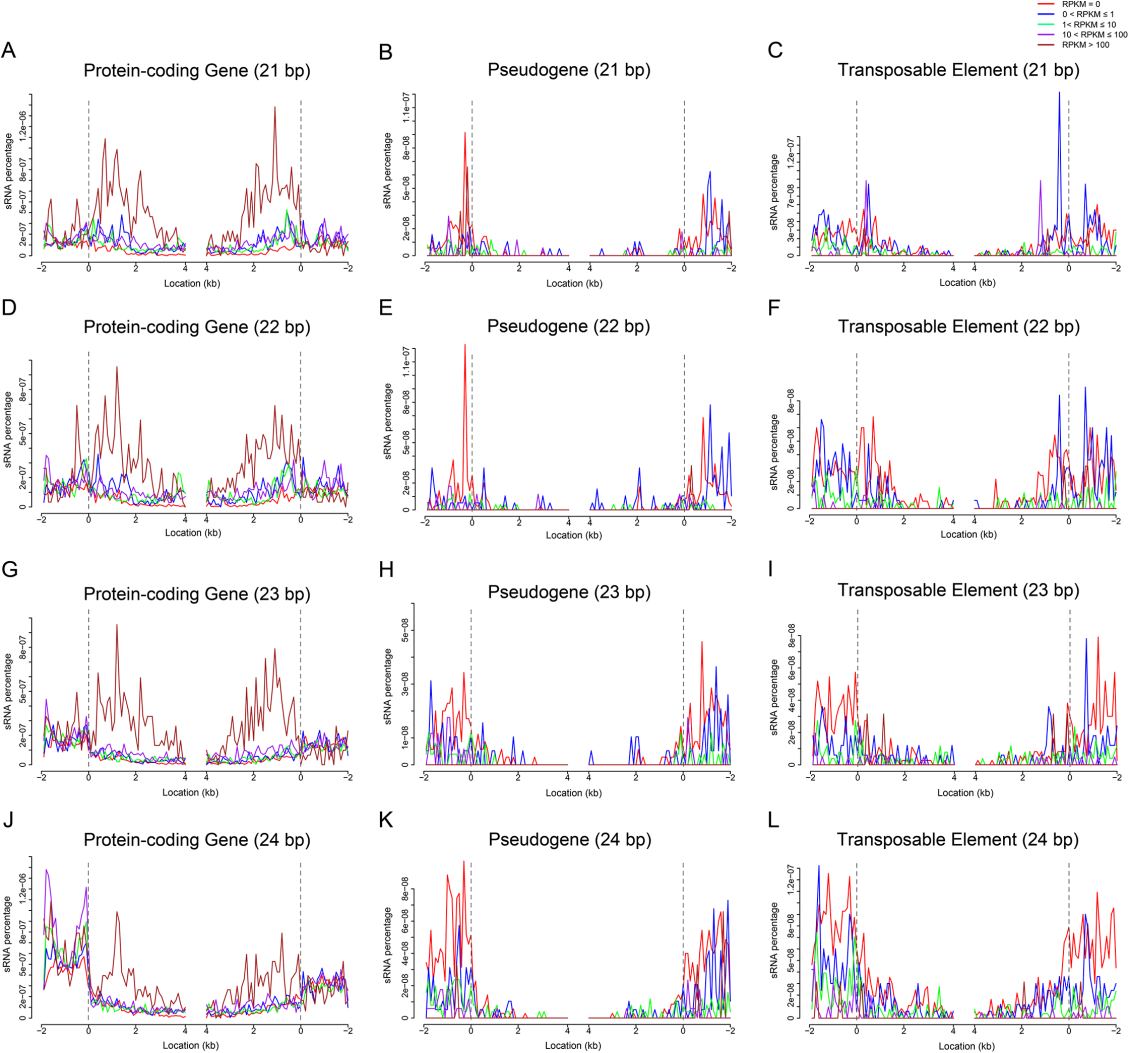
sRNA associated with gene expression in the embryo. (A to L) show 21-24-nt sRNAs that regulate protein-coding gene expression [(A), (D), (G) and (J)], pseudogene gene expression [(B), (E), (H), and (K)] and transposable element activity [(C), (F), (I),and (L)]; 21-nt sRNAs are shown in (A), (B), and (C), 22-nt sRNA in (D), (E), and (F), 23-nt sRNA in (G), (H), and (I), and 24-nt sRNA in (J), (K), and (L). Gene expression was classified into five levels according to the number of reads per kilobase per million reads (RPKM, see Materials and Methods), and the correlation between sRNA accumulation and gene expression was investigated. The dashed line at zero represents the point of alignment. Note that there are only 13 pseudogenes and 20 TEs whose RPKM value is higher than 100 in the embryo, meaning that the sample size was too small to be statistically significant.

siRNAs regulate gene expression through directing DNA methylation or degrading mRNAs [8, 27]. In maize outer layer of mature ear prior to fertilization, the 24-nt siRNAs accumulated at gene ends [28]. In our dataset, all of the sRNAs ranging from 21 nt to 24 nt in length accumulated predominantly at the ends of protein-coding genes and in the upstream or downstream regions of TEs and pseudogenes both in embryo and endosperm (Fig 4; Fig D-J in S1 File).

We asked whether sRNAs production is associated with gene expression. The protein-coding genes and pseudogenes and TEs were grouped into five levels by expression (see “Materials and Methods”), and a genome-wide association of sRNA accumulation with gene expression was performed in both the embryo and endosperm (Fig 4; Fig J in S1 File). In the transcripts of protein-coding genes, significant accumulation of 21-24-nt sRNAs was detected mainly in genes with high levels of expression (RPKM > 100; Fig 4A, 4D, 4G and 4J; Fig J in S1 File). However, in TEs and pseudogenes, high accumulation of sRNAs was detected mainly in genes with low expression (Fig 4B, 4C, 4E, 4F, 4H and 4K; Fig J in S1 File).

### The association of DNA methylation with gene expression

**Fig 5:**
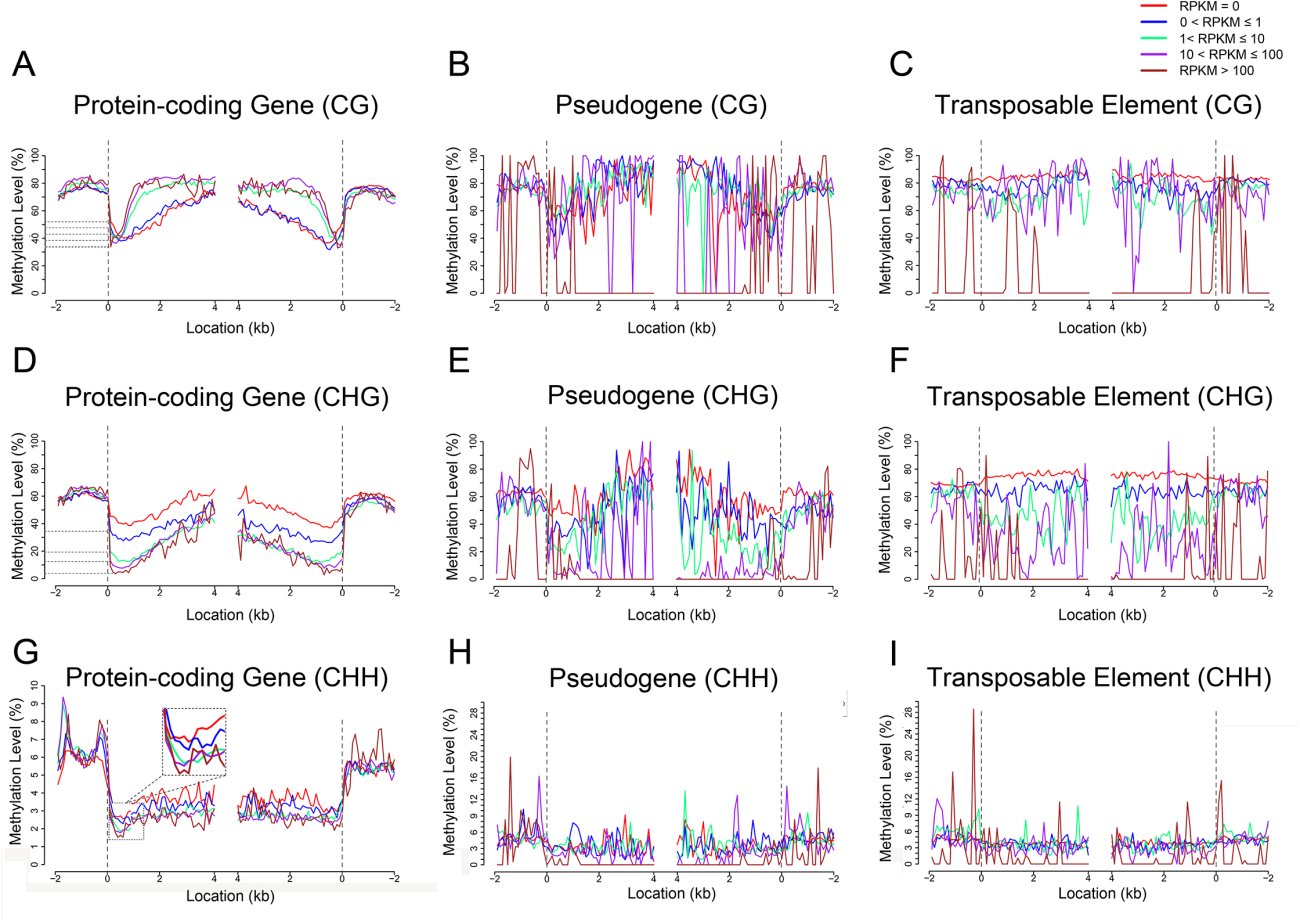
DNA methylation associated with gene expression in the embryo. (A to I) The relationships between DNA methylation and protein-coding gene expression [(A), (D), and (G)], pseudogene gene expression [(B), (D), and (H)] and transposable element activity [(C), (F), and (I)]; CG methylation is shown in (A), (B), and (C), CHG in (D), (E), and (F), and CHH in (G), (H), and (I). The dashed line at zero represents the point of alignment. As shown in Fig 4, the number of pseudogenes and TEs with RPKM>100 are only 13 and 20 in the embryo, respectively.

Cytosine methylation plays important roles in regulating gene expression and TE silencing in plants and animals [29–33]. To understand the relationship between cytosine methylation and gene expression in maize seeds, we evaluated correlations of mRNA-seq data with methylC-seq data (see “Materials and Methods”). The effects of methylation on gene expression were sequence context- or gene-dependent. CG methylation in transcribed regions seemed to be positively correlated with the expression level, whereas CHG methylation negatively correlated, suggesting an opposite role in gene expression regulation between CG and CHG methylation. Interestingly, protein-coding gene expression varied inversely with CG, CHG, or CHH methylation around the TSS (Transcriptional Start Site) or TTS (Transcriptional Terminal Site) (Fig 5A, 5D and 5G). For example, it’s evident that genes with highest abundance of transcripts (RPKM > 100) at TSS or TTS had lowest CHG methylation level; in contrast, genes with lowest abundance of transcripts (RPKM = 0) had highest CHG methylation level (Fig 5D). Another interesting observation was the presence of two CHH islands, which exhibited high density of CHH methylation, within 2-kb upstream of protein-coding genes, and CHH methylation in the TSS-proximal CHH island was positively correlated with transcription (Fig 5G). In addition, the correlation between methylation at TSS and TTS regions with transcription was also observed in pseudogenes, albeit it was not as high as that in protein-coding genes (Fig 5B and 5E).

To further demonstrate the relationship between gene expression pattern and methylation status, two particular genes, *ZmFie1* and *floury-1*, were chosen from the dataset and characterized. *ZmFie1* is one of the maize imprinted genes which shares high levels of similarities to Drosophila Polycomb-group genes. Previous studies demonstrated that Arabidopsis *Fie1* gene, the ortholog of maize *Fie1*, was specifically expressed in the endosperm tissue [34–36]. *floury-1* which shows parent-of-origin phenotypes, is a potential imprinting gene [37]. In our study, both *Fie1* and *floury-1* were found to be specifically expressed in the maize endosperm (Table E in S1 File), and the DNA methylation levels of both genes in embryo were significantly higher than that in endosperm at all kinds of sequence contexts (Fig K in S1 File).

**Fig 6:**
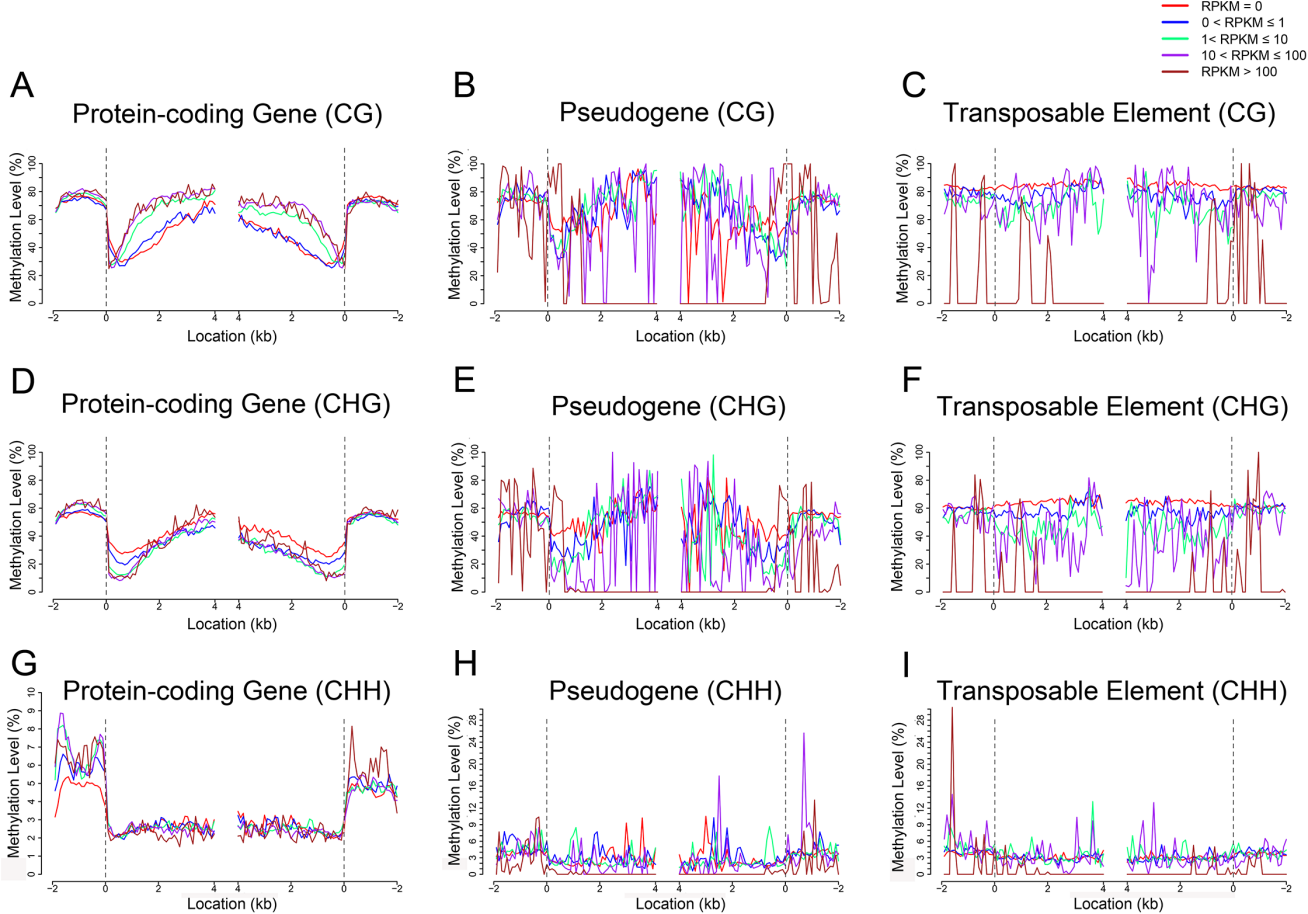
DNA methylation associated with gene expression in the endosperm. (A to I) show the relationships between DNA methylation and protein-coding gene expression [(A), (D), and (G)], pseudogene gene expression [(B), (D), and (H)] and transposable element activity [(C), (F), and (I)]; CG methylation is shown in (A), (B), and (C), CHG in (D), (E), and (F), and CHH in (G), (H), and (I). The dashed line at zero represents the point of alignment. As shown in Fig J in S1 File, the number of pseudogenes and TEs with RPKM>100 are only 14 and 15 in the embryo, respectively.

TEs were opposite to protein-coding genes regarding the effects of CG methylation on gene expression, as demonstrated by the observation that TEs with low expression showed high levels of CG methylation evenly across entire regions from upstream to downstream (RPKM < 1; Fig 5C; Fig 6C). Similar effects of CHG or CHH were also observed for the TEs with low expression level (RPKM < 1; Fig 5F and 5I; Fig 6F and 6I). These observations indicated that expression of protein-coding genes and TEs may be differentially regulated by DNA methylation. In addition, high level of DNA methylation within pseudogenes at CHG or CHH context led to low expression (Fig 5E and 5H; Fig 6E and 6H).

## Discussion

In this study, we used next-generation sequencing technology to identify single-base DNA methylome, transcriptome and smRNAome in maize seeds at early developing stage. High throughput analysis of these data deciphered a complex landscape of gene expression profiling regulated by cytosine methylation and sRNAs.

DNA methylation, an epigenetic modification, has been found in diverse eukaryotic organisms and plays a key role in embryogenesis, genomic imprinting, and tumorigenesis in mammals, and in transposon silencing and gene regulation in plants [32, 38–42]. The single-base cytosine methylation maps of some organisms, including human [24], Arabidopsis [23, 25], rice [21], silkworm [43], and tomoto [44] have been reported. During the preparation of the manuscript, single-base DNA methylation sequencing of the outer layer of mature maize ears prior to fertilization was reported [28]. We sequenced the 9-DAP maize embryo and endosperm DNA methylome using the bisulfite-based whole-genome sequencing. Like rice and Arabidopsis, the maize endosperm is hypomethylated compared to the embryo, and the CG methylation pattern is highly similar amongst the three plant species [21, 23]. However, some drastic differences in CHG and CHH methylation between the different species were observed. For example, both rice and Arabidopsis gene bodies contained almost exclusively CG methylation, whereas maize contained not only CG but also CHG methylation [21, 23], implying that maize genome may have evolved a more complex regulatory mechanism underlying protein-coding gene expression than rice and Arabidopsis. The single-base resolution of bisulfite-Seq technology allows determination of the precise boundaries between methylated and unmethylated regions. For example, we observed that the boundary between repeats and flanking DNA showed an apparent peak of CHH methylation, which was not detected in other DNA sequences including protein-coding genes, pseudogenes and TEs (Fig 1F). This apparent peaking methylation was correlated with sRNA accumulation (Fig 3F; Fig G-I in S1 File), suggesting that the CHH methylation in the boundary regions is probably regulated by sRNAs through the RdDM pathway.

DNA methylation repressed gene expression by blocking transcription factors binding to the promoters. High methylation levels of promoters are correlated with low or no transcription [45]. However, this was not found in the maize seeds: the transcript abundance in protein-coding genes was not correlated with the DNA methylation of the promoters. Early days of DNA methylation research on human revealed that transcribed genes are featured with gene body methylation [46]. Thereafter, extensive studies have illustrated positive correlations between transcription and gene body methylation in plant and animal genomes [23, 24, 47]. We found that CG and CHG as well as CHH methylation within gene body all influenced transcription: high level of CG methylation or low level of CHG or CHH methylation was corresponding to active transcription (Fig 5A and 5D; Fig 6A and 6D), indicating that CG methylation of gene body may stimulate transcription elongation, whereas CHG/CHH methylation of gene body may block transcription elongation. Rice methylation patterns closely resemble those of Arabidopsis in many salient features: modestly expressed genes are most likely to be methylated [21, 32, 42]. In contrast, inactive genes exhibited high levels of CHG/CHH methylation in maize (Fig 5D and 5G). Previously it was also reported that in cancer cells there existed genome-wide inverse relationship between methylation of non-CG islands and expression [48]. Taking into account all the studies performed in variety of eukaryotes, it can be concluded that gene body methylation other than promoter methylation is an ancient property of the genomes, and transcription elongation seems to be under opposite control by CG and CHG/CHH methylation, respectively, in maize seeds.

It is demonstrated that CG methylation around the TSS and TTS negatively affects gene expression in rice [21]. In this study, we found that not only CG methylation but also CHG/CHH methylation of the TSS- or TTS-proximal regions were inversely correlated with gene expression (Fig 5; Fig 6). This suggests that lack of methylation in both TSS and TTS is important for gene expression, and it’s likely that the epigenetic mechanisms underlying gene expression are more complex in maize than in rice. Previous studies reported that methylated CG islands at TSSs cannot initiate transcription after the DNA has been assembled into nucleosomes which are the substrates for *de novo* methylation [49–51]. It can also be concluded that in maize both transcription initiation and transcription termination seem to be sensitive to DNA methylation silencing. In addition, functioning of CG methylation varies with the position where CG methylation occurs: within gene body CG methylation may play a stimulating role in the regulation of gene expression, and methylation at TSS or TTS CG may negatively influence gene expression. Very recently it was reported that major classes of transposons close to cellular genes exhibited a peak of CHH methylation in maize, which was named CHH islands [28]. Likewise, two peaks of CHH methylation were found in the 9-DAP maize seeds in this study (Fig 5G). The difference in the number of CHH islands may reflect the dynamics of CHH methylation in different tissues or different development stage. We observed a positive correlation between methylation in CHH islands and gene transcription, indicating that genes with high transcription intend to confer high CHH methylation to the intergenic regions close to genes.

It is estimated that the maize genome contain more than 10,000 pseudogenes [16]. Three classes of pseudogenes have been identified: nonprocessed pseudogenes derived from gene duplication, processed pseudogenes originated from retrotransposition, and unitary pseudogenes arising from spontaneous mutations in protein-coding genes [52–54]. Since pseudogenes are generally noncoding, they are considered “junk DNA” [55, 56]. Even though recent studies indicate that the pseudogenes have diverse functions to regulate their parental gene expression or unrelated genes [53], the regulatory mechanism of pseudogenes remains unclear. Nevertheless, the methylation level of pseudogenes was higher than genes and repeats in Arabidopsis [32, 42]. Our data also demonstrated that the level of methylation in pseudogenes was higher than that in protein-coding genes in maize seeds (Fig 1), suggesting a possible link between the enhanced DNA methylation and loss of protein coding. Moreover, the observation that the methylation profiles of protein-coding genes and pseudogenes are similar in shape is suggestive of a common epigenetic mechanism governing the transcription of protein-coding genes and pseudogenes (Fig 1). How the DNA methylation and sRNAs interact to regulate pseudogene expression needs to be further elucidated.

siRNAs cause RNA-directed DNA methylation. Previous studies demonstrated that only a fraction of the siRNA clusters (i.e. endogenous loci corresponding to high local concentrations of siRNAs) are heavily methylated in Arabidopsis, suggesting that a large amount of DNA methylation is maintained without persistent targeting by siRNAs [42]. In this study we found that there were no correlations between CG/CHG methylation and sRNA accumulation, but in some positions (i.e. upstream regions of protein-coding genes and pseudogenes) higher accumulation of 24-nt sRNAs corresponded to denser methylation (Fig 3). This is consistent with the previous reports. We also observed that although higher abundance of sRNAs,was present in the upstream region of TEs as compared to TEs themselves, but CHH methylation occurred evenly from upstream to downstream. This may be due to the fact that TEs were not grouped for the correlation analysis by their proximity to cellular genes [28]. Small RNAs and DNA methylation interacted to induce the silencing of TEs [57]. In maize embryo and endosperm, high level of DNA methylation corresponded to the low TE expression (Fig 5; Fig J in S1 File), and high level of sRNA accumulation in the upstream or downstream of TEs corresponded to low expression (Fig 4; Fig I in S1 File). However, the DNA methylation level is not consistent with the sRNA accumulation (Fig 3; Fig C-H in S1 File). These results indicate that sRNA and DNA methylation may repress TE expression through different mechanisms in maize.

In summary, maize embryos and endosperm on DAP 9, an important developmental stage featured by starting filling, were intensively characterized for the relationship of cytosine methylation with transcription expression on a genome-wide scale using high throughput sequencing technology. The data suggest that maize has evolved more complex epigenetic machinery than rice and Arabidopsis, and different DNA context methylation has different role in gene expression regulation. Moreover, the mode of methylation-regulated gene expression varies with gene type, sequence context or position of a given gene. However, it’s necessary to point out that the relationship of DNA methylation with gene transcription in maize seeds characterized in this study was only of a one time-point, not necessarily representing that in other tissues or other developmental stages given the dynamics of DNA methylation. A comprehensive understanding of the effects of DNA methylation on gene expression in maize seeds awaits further investigation of the whole developmental process.

## Materials and Methods

### Plant material

The maize inbred line B73 was grown in the field during the summer of 2009 in Langfang, Hebei province, China. The field where we conducted the experiment belongs to Biotechnology Research Institute, Chinese Academy of Agricultural Sciences. Ears were bagged before silk emergence. Each set of inbred kernels were generated on the same day by self-pollination. On 9^th^ day after pollination (DAP), the endosperm and embryo were isolated using tweezers and collected in 300 mM sorbitol solution with 5 mM MES (pH 5.7) from the ovules, and were then transferred into tubes, snap-frozen in liquid nitrogen and stored at -80°C for further use. The batch of seed samples used in this study is the same as that described in our previous study [18].

### MethylC-Seq library generation

Genomic DNA (10 μg) was extracted from the embryo and endosperm using the DNeasy Mini Kit (Qiagen). The DNA was fragmented by sonication to 280–350 nt with a Bioruptor (Diagenode). The DNA was end-repaired using a mixture of T4 DNA polymerase, Klenow DNA polymerase and T4 PNK (Enzymatics), and a 3’ overhang A was added using the Klenow exo-enzyme (Enzymatics). The resultant fragments were ligated with the Illumina methylation adapters by DNA T_4_ ligase (Enzymatics) according to the Illumina protocol. Adapter-linked DNA fragments were bisulfated using the EZ DNA Methylation Kit (Zymo), as per the manufacturer’s protocol. The treated DNA was amplified by PCR for 11 cycles. The DNA fragments were purified, quantified and then sequenced for 100 cycles using the Illumina protocol.

### RNA-Seq library generation

Total RNA (10 μg) from each sample was extracted using RNeasy Mini Kit (Qiagen), according to the manufacturer’s protocol. mRNA was isolated from total RNA using 7 μl of oligo dT on Sera-magnetic beads and 50 μl of binding buffer. mRNA was fragmented by metal hydrolysis in RNA fragment buffer (Ambion) for 2 min at 70°C. The reaction was stopped by adding 2 μl of fragmentation stop solution (Ambion). The fragmented RNA was converted to double-stranded cDNA. After polishing the ends of the cDNA, an adenine base was added at the 3’ ends, after which Illumina multiplex adaptors were ligated. The ligated DNA was separated on 2% agarose gel and 300-nt targeted DNA was extracted. DNA was purified from the gel using the Qiagen Gel extraction kit. The purified DNA was amplified by 15 cycles of PCR, and the PCR DNA was then purified on the Qiagen PCR purification kit to obtain the final seq library for sequencing. The DNA concentration of the seq library was determined on Qubit (Invitrogen).

### sRNA library generation

Total RNA (10 μg) from each sample was extracted using the RNeasy Mini Kit (Qiagen) according to the manufacturer’s protocol. Novex 15% TBE-Urea gel (Invitrogen) was used to isolate small RNA fragments (30 nt in length) from total RNA. The purified small RNAs were ligated to a 5’ adaptor (Illumina) and the ligation products were purified in Novex 15% TBE-Urea gels. Next, a 3’ adaptor (Illumina) was ligated to the 5’ ligation products and further purified in a Novex 10% TBE-Urea gel (Invitrogen). Reverse transcriptase PCR was used to reverse transcribe these ligation products. Then, a 6% TBE-Urea gel (Invitrogen) was used to purify the amplification products. The DNA fragments were purified, quantified and then sequenced for 36 cycles using the protocol provided by Illumina.

### High-throughput sequencing

MethylC-Seq, RNA-Seq and sRNA-seq libraries were sequenced using the Illumina HiSeq 2000, as per the manufacturer’s protocol. The paired-end protocol was used for RNA-Seq sequencing, while the single ends sequencing dataset was used for MethylC-Seq sequencing. Read lengths of RNA-seq and MethylC-Seq were up to 100 nt. Image analysis and base calling were performed with the standard Illumina pipeline.

### MethylC-Seq analysis

The raw data in FastQ format produced by the Illumina pipeline were first pre-processed, including: a) Filtering of low quality reads and b) trimming reads to before the first occurrence of a low-quality base (quality score < 20). Remaining short sequences were mapped to the maize reference genome (RefGen ZmB73 Release 5b) using Bismark version 0.4.1 [58], allowing up to four mismatches per read. Only uniquely aligning reads were retained for the next procedure. Three types of methylation calls (CG, CHG, CHH), which were covered by at least 10 reads excluding any duplication, were extracted. For each sequence context, bulk fractional methylation were calculated using the formula #C/(#C+#T). Fractional methylation within a 50-nt sliding window was also calculated to identify the differential methylation region (DMR) between the maize endosperm and embryo. The upstream two nucleotides and downstream four nucleotides surrounding cytosines were analyzed to determine whether they have local sequence effects on DNA methylation of the CG, CHG, and CHH contexts. The annotations of genes, repeat regions, transposable elements and pseudogene regions were retrieved from the B73 filter gene set (release 5b).

### RNA-Seq analysis

RNA-seq datasets were aligned to the maize reference genome using tophat [59]. The resulting alignment files were subjected to Cufflinks [60] to generate a transcriptome assembly and make the annotation. Reads per kilobase of transcript per million reads (RPKM) were calculated. Five ranges of RPKM values representing different expression levels were collected and associated with DNA methylation and sRNA accumulation.

### sRNA-Seq analysis

Read sequences produced by the Illumina analysis pipeline were mapped to the maize reference sequence using bwa [61]. Up to two mismatches were allowed in the alignment. Information from the B73 filter gene set release 5b was used to make the annotation. sRNAs were then separated according to length (21 to 24 nt) to identify the accumulation at different regions. sRNAs of specific lengths were normalized (divided by the total number of sRNAs), and the sRNA percentage (2 kb distal from to 4 kb into the gene) for each 100-nt interval was calculated.

### Sequence Data

The data for this article have been deposited at the National Center for Biotechnology Information under accession number SRP056646.

## Supporting Information

S1 File Fig A, Distribution of the percentage methylation in the CG, CHG and CHH contexts. The y axis indicates the fraction of the total methylcytosines that display each percentage of methylation (x axis), defined as the fraction of reads at a reference cytosine containing cytosines following bisulfite conversion. Fractions were calculated within bins of 20%, as indicated on the x axis. Fig B, DMR distributions of repeats, transposable elements, pseudogenes, and protein-coding genes in the embryo and endosperm. (A to C) DMR distributions in repeats of type I transposons (LTR and LINE) and type II transposons (TIR). (D to F) represent the DMR distributions in TEs, pseudogenes and protein-coding genes, respectively. Fig C, Local sequence effects on DNA methylation in the endosperm. Sequence contexts that are preferentially methylated in the endosperm for 7-mer sequences, in which the methylated cytosine is in the third position. (A), CG context; (B), CHG context; (C), CHH context. The y axis indicates the methylation level, and the x axis indicates the base composition and position. Fig D, Correlation between 21-nt sRNA and DNA methylation. (A to D) indicate the correlations between 21-nt sRNA and DNA methylation in protein-coding genes (A), repeats (B), TEs (C) and pseudogenes (D). The dashed line at zero represents the point of alignment. Fig E, Correlation between 22-nt sRNA and DNA methylation. (A to D) indicate the correlations between 22-nt sRNA and DNA methylation in protein-coding genes (A), repeats (B), TEs (C) and pseudogenes (D). The dashed line at zero represents the point of alignment. Fig F, Correlation between 23-nt sRNA and DNA methylation. (A to D) indicate the correlations between 23-nt sRNA and DNA methylation in protein-coding genes (A), repeats (B), TEs (C) and pseudogenes (D). The dashed line at zero represents the point of alignment. Fig G, Correlation between 21-nt sRNA and CHH methylation. (A to D) indicate the correlations between 21-nt sRNA and CHH methylation in protein-coding genes (A), repeats (B), TEs (C) and pseudogenes (D). The dashed line at zero represents the point of alignment. Fig H, Correlation between 22-nt sRNA and CHH methylation. (A to D) indicate the correlations between 22-nt sRNA and CHH methylation in protein-coding genes (A), repeats (B), TEs (C) and pseudogenes (D). The dashed line at zero represents the point of alignment. Fig I, Correlation between 23-nt sRNA and CHH methylation. (A to D) indicate the correlations between 23-nt sRNA and CHH methylation in protein-coding genes (A), repeats (B), TEs (C) and pseudogenes (D). The dashed line at zero represents the point of alignment. Fig J, sRNA associated with gene expression in the endosperm. (A to L) show that 21-24-nt sRNAs regulate protein-coding gene expression [(A), (D), (G) and (J)], pseudogene gene expression [(B), (E), (H), and (K)] and TE activity [(C), (F), (I), and (L)]; 21-nt sRNAs are shown in (A), (B), and (C), 22-nt sRNAs in (D), (E), and (F), 23-nt sRNAs in (G), (H), and (I), and 24-nt sRNAs in (J), (K), and (L). The dashed line at zero represents the point of alignment. Note that there are only 14 pseudogenes and 15 TEs whose RPKM value is above 100 in the embryo, meaning that the sample size was too small to be statistically significant. Fig K, DNA methylation patterns of *Fie1* and *floury-1*. (A) DNA methylation pattern of *Fie1*. (B) DNA methylation pattern of *floury-1*. Table A, Statistics of DNA methylation in embryo and endosperm. Table B, Methylation fraction distribution in embryo and endosperm. Table C, DMR distribution in Embryo and Endosperm. Table D, Statistics of DMR between embryo and endosperm. Table E, Gene expression in embryo and endosperm. (PDF)

S1 Table Sequence preferences for methylation. (XLS)
