# Supplementary material for: Genome-Wide Epigenetic Regulation of Gene Transcription in Maize Seeds
Source: PLoS One. 2015 Oct 15;10(10):e0139582. doi: 10.1371/journal.pone.0139582 (PMC4607434; doi:10.1371/journal.pone.0139582)
Supplement: S1 File — (PDF) [file pone.0139582.s001.pdf]

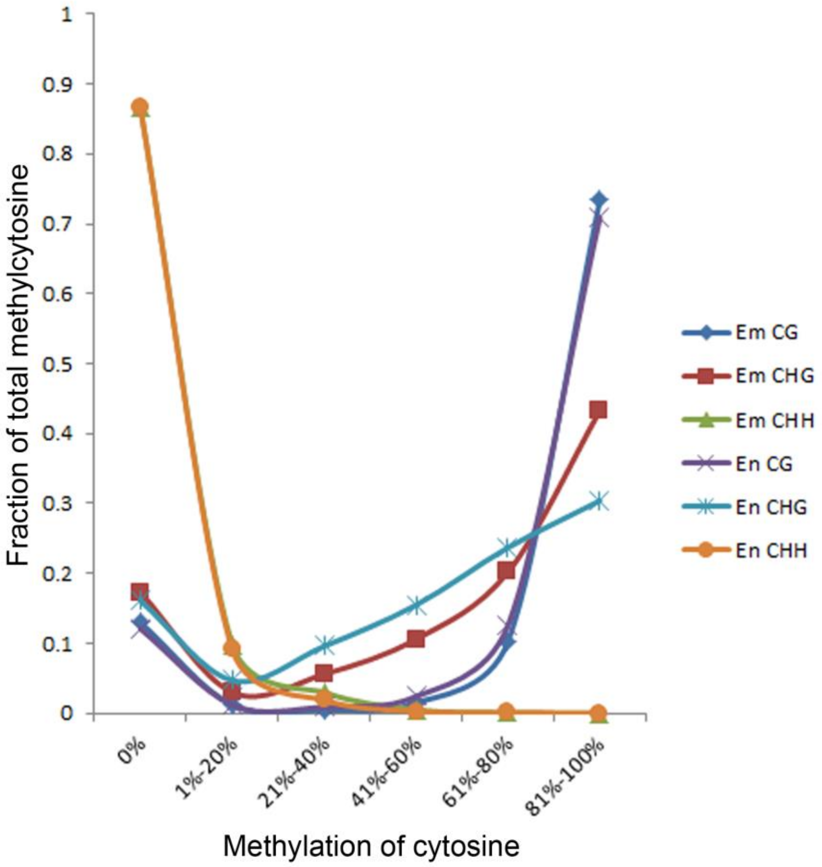

Figure A. Distribution of the percentage methylation in the CG, CHG and CHH contexts.

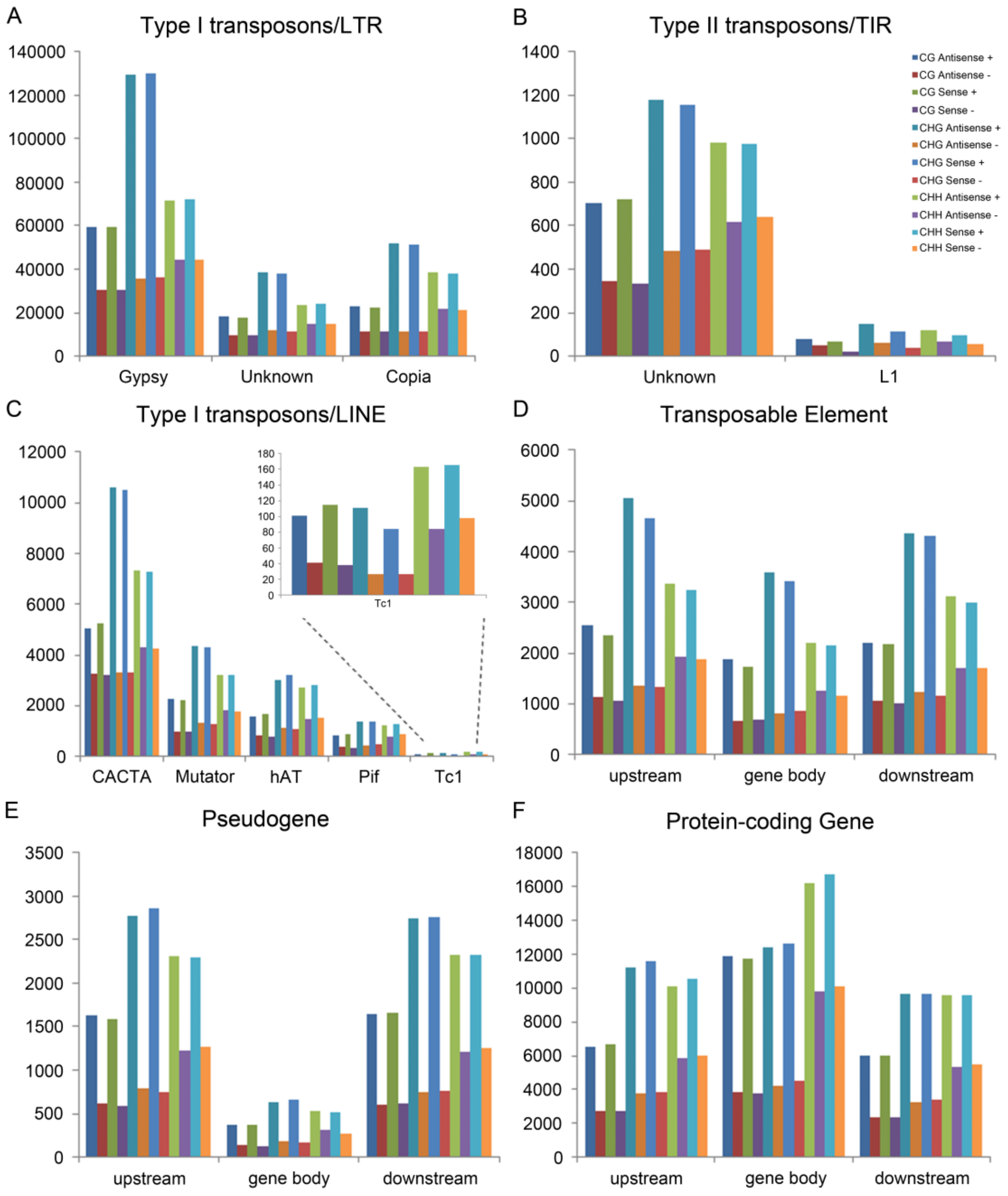

Figure B DMR distributions of transposons, transposable elements, pseudogenes, and protein-coding genes in the embryo and endosperm.

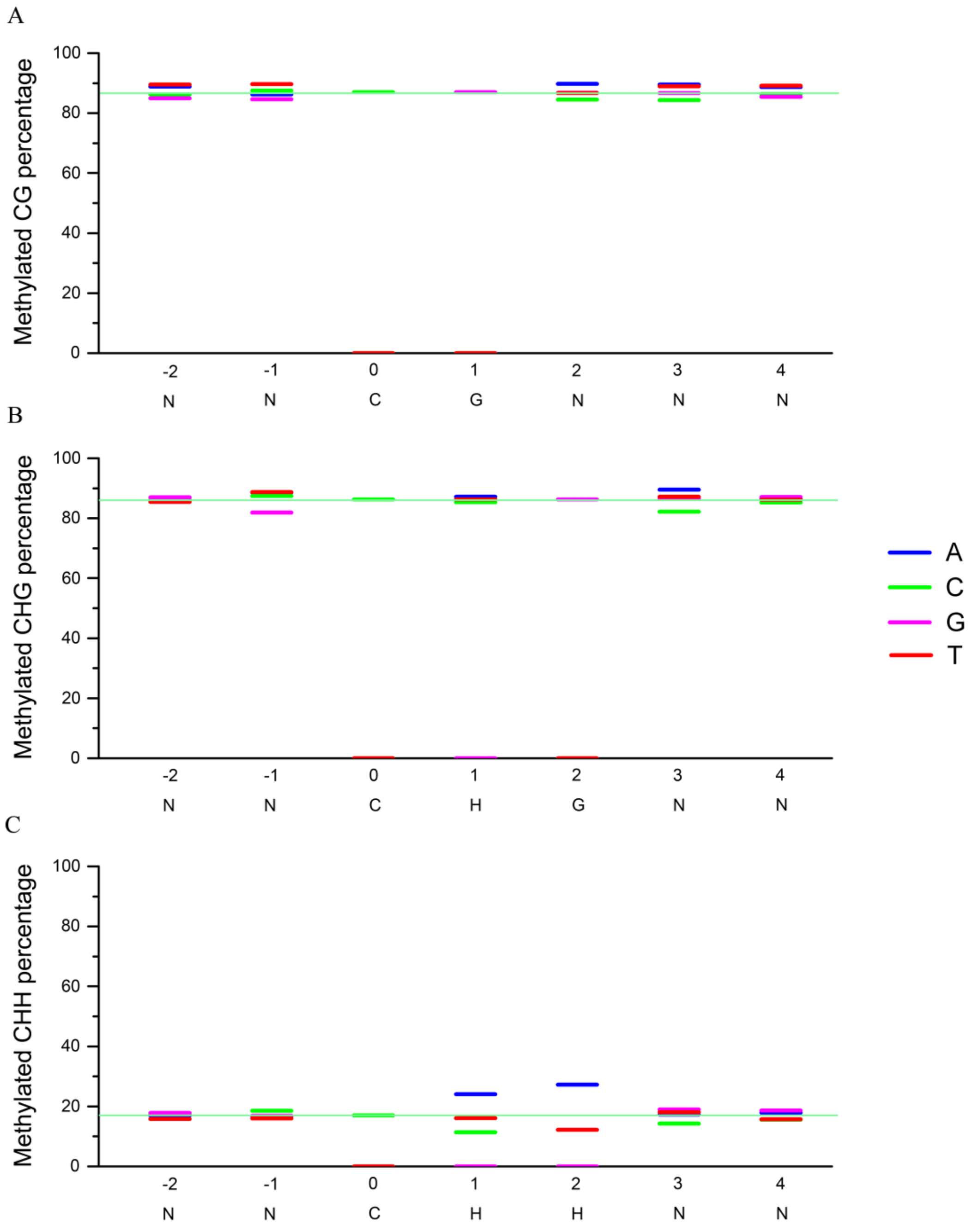

Figure C. Local sequence effects on DNA methylation in the endosperm.

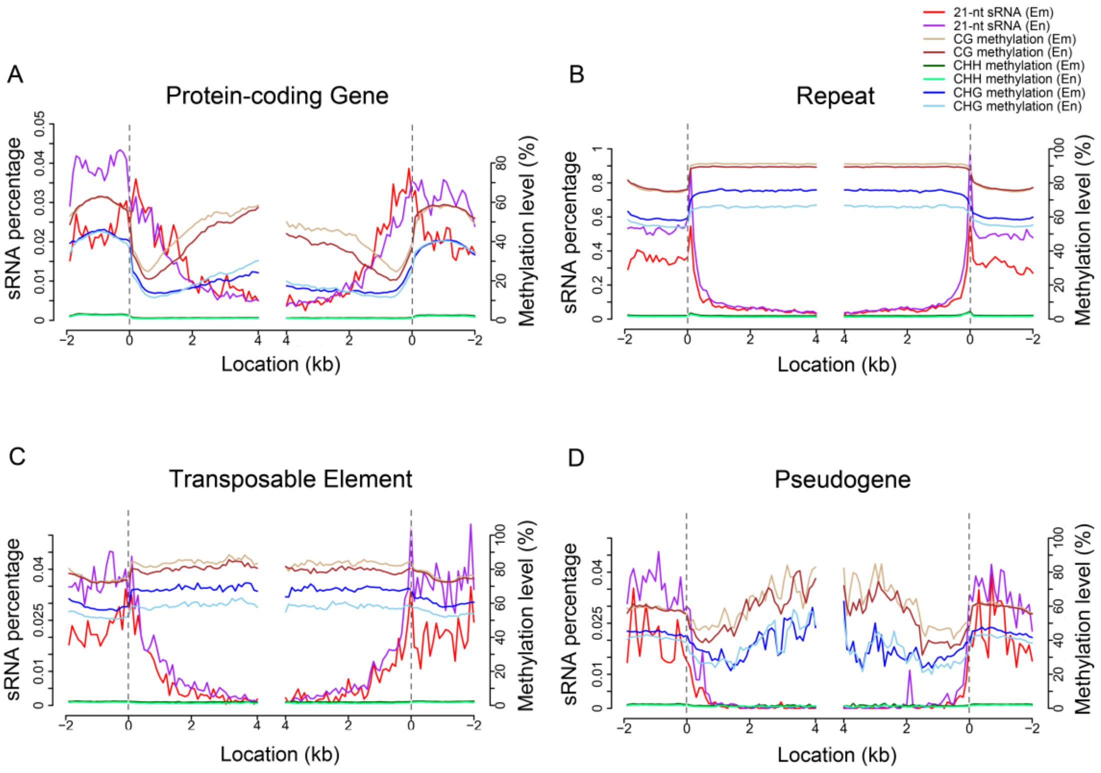

Figure D. Correlation between 21-nt sRNA and DNA methylation.

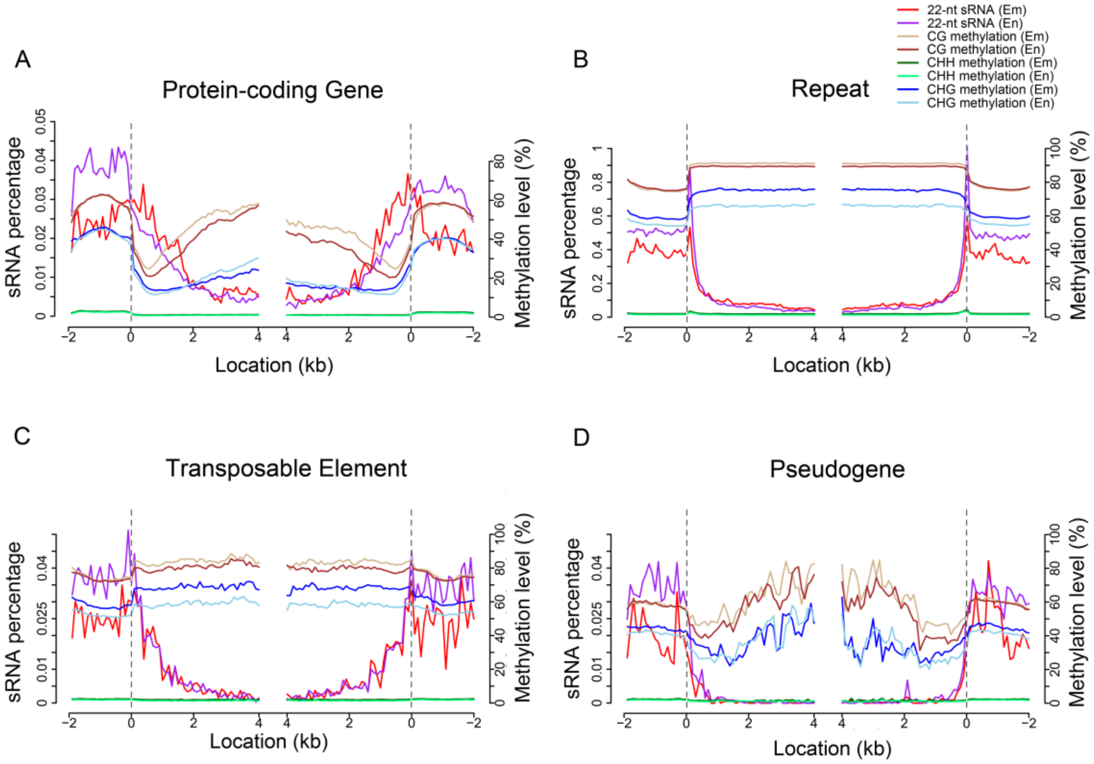

Figure E. Correlation between 22-nt sRNA and DNA methylation.

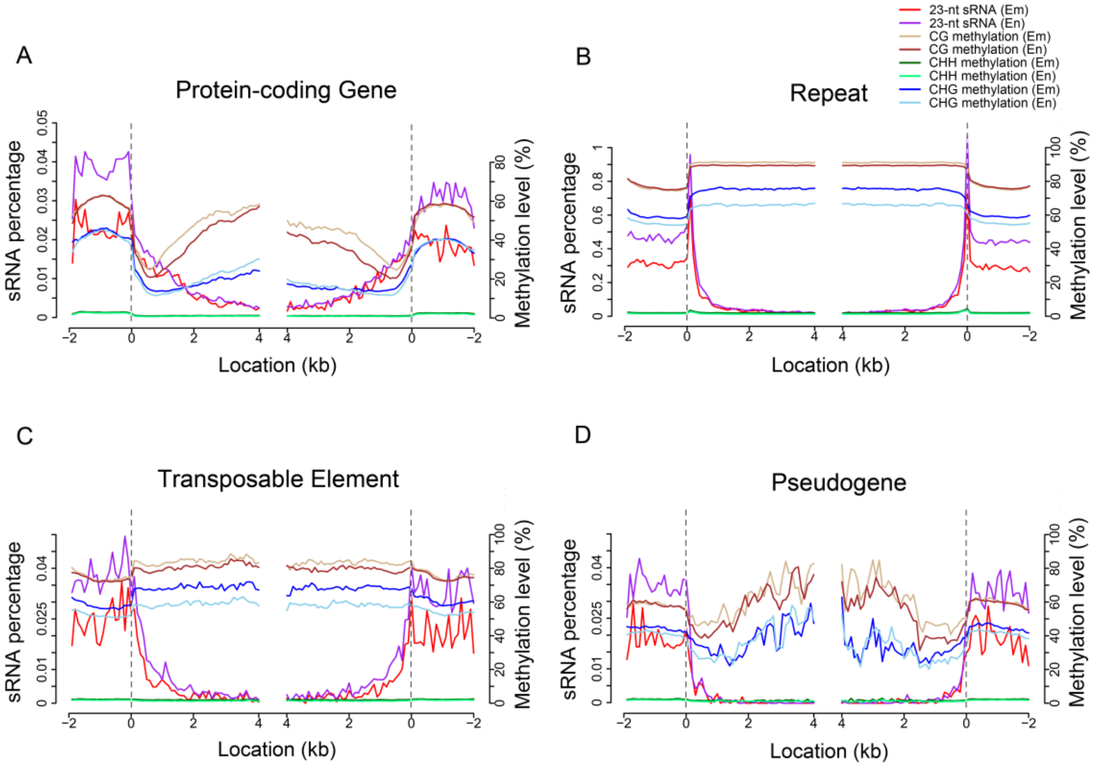

Figure F. Correlation between 23-nt sRNA and DNA methylation.

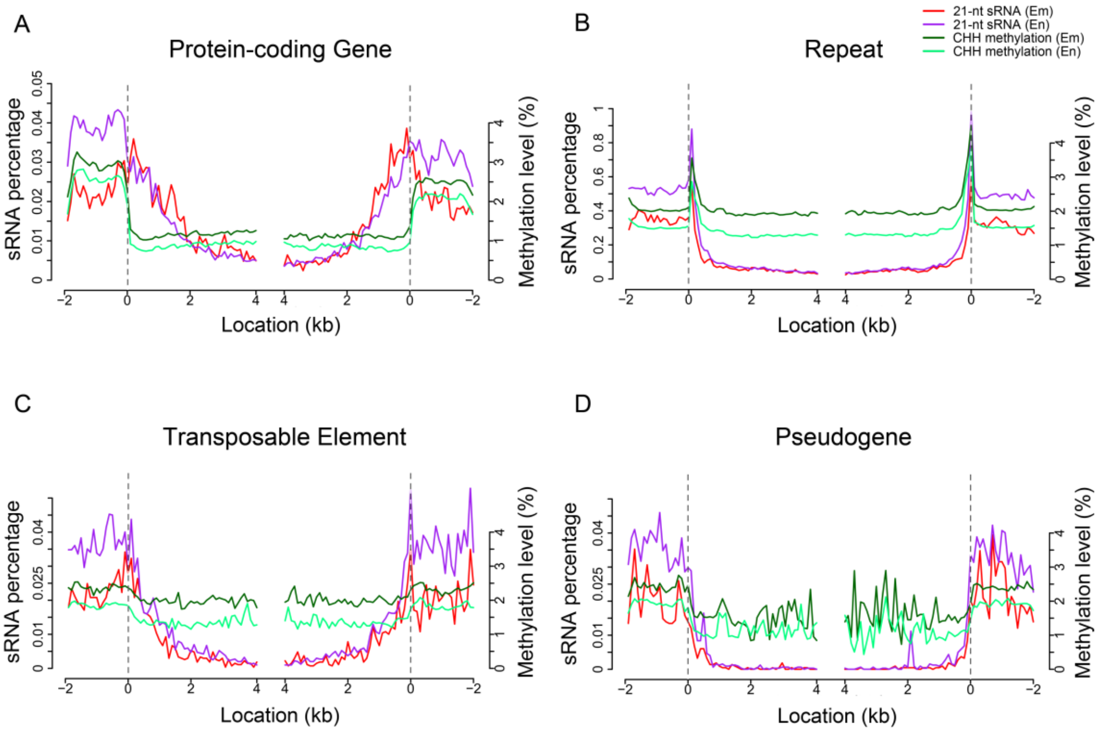

Figure G. Correlation between 21-nt sRNA and CHH methylation.

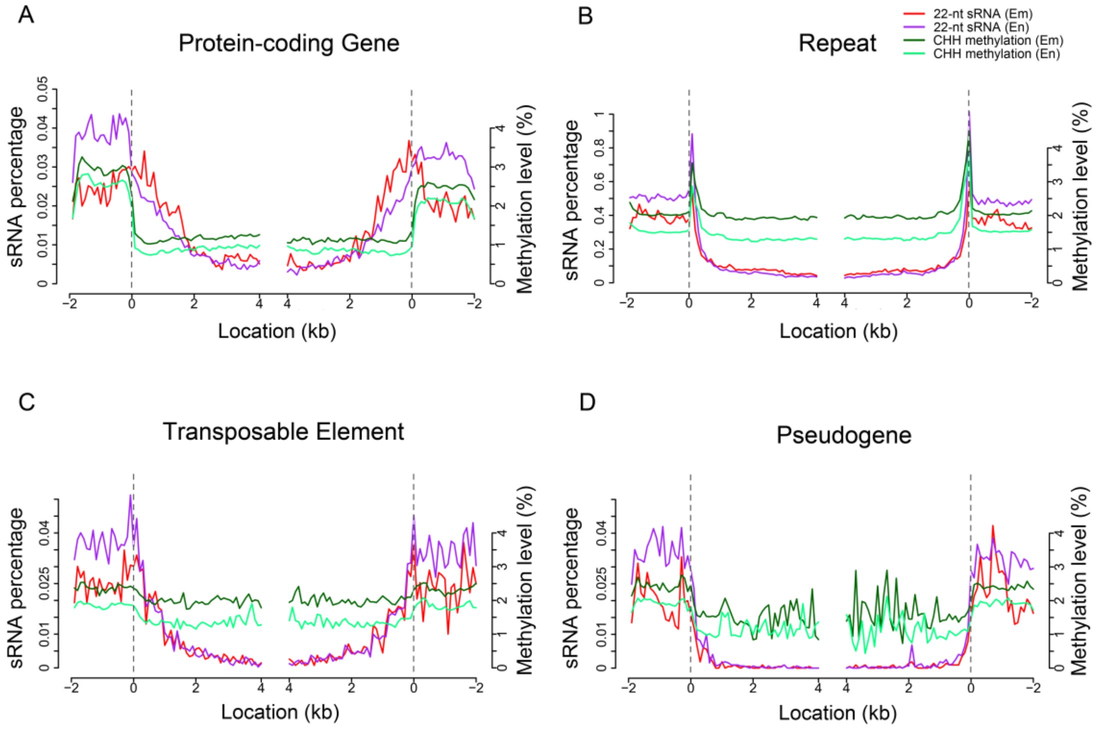

Figure H. Correlation between 22-nt sRNA and CHH methylation.

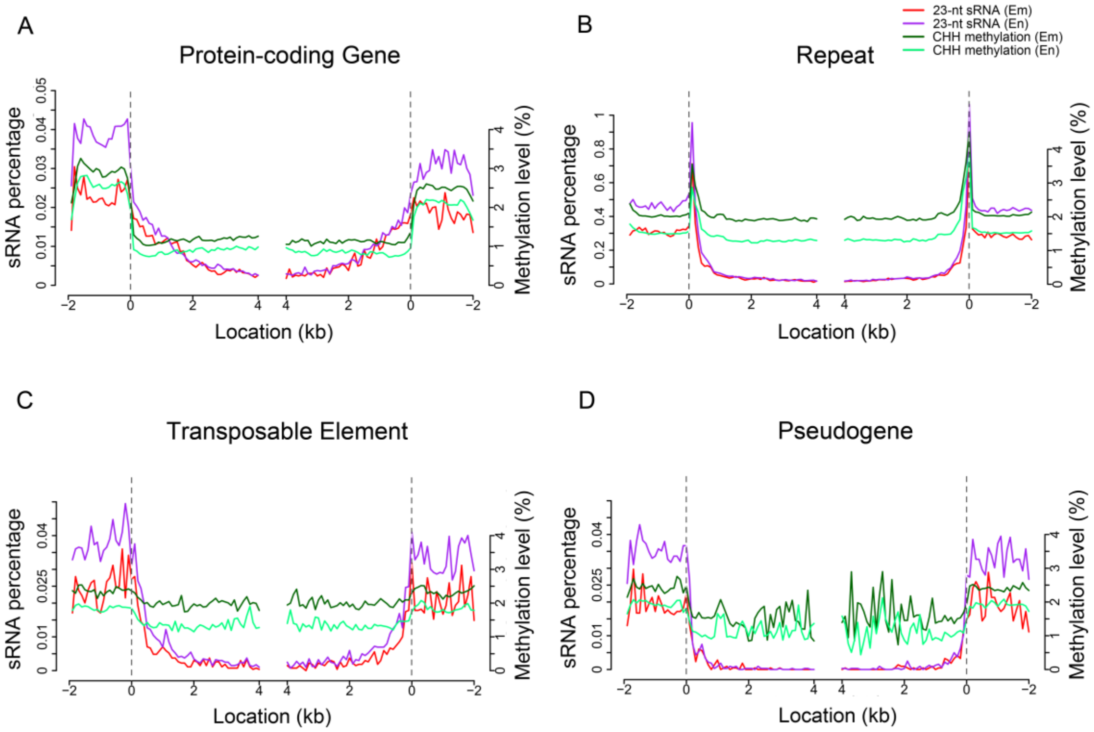

Figure I. Correlation between 23-nt sRNA and CHH methylation.

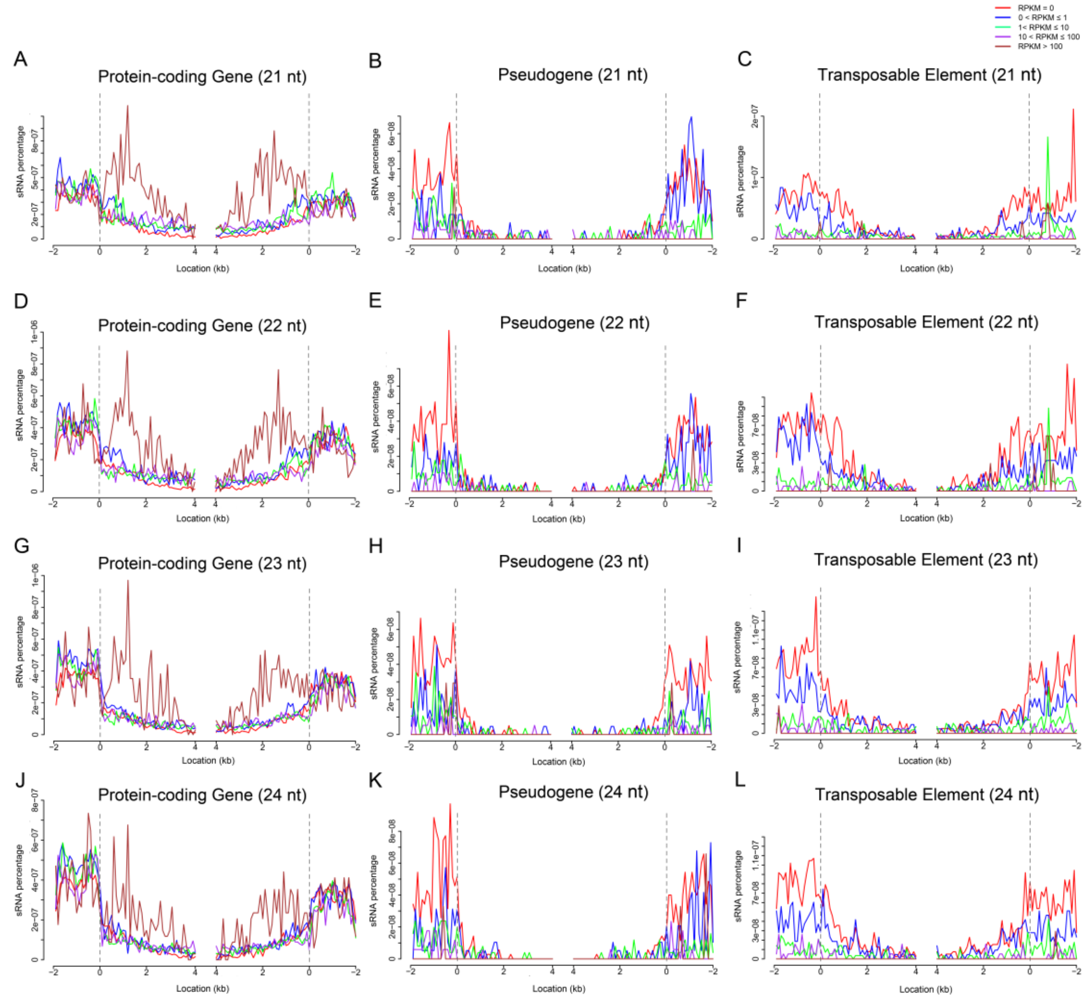

Figure J. sRNA associated with gene expression in the endosperm.

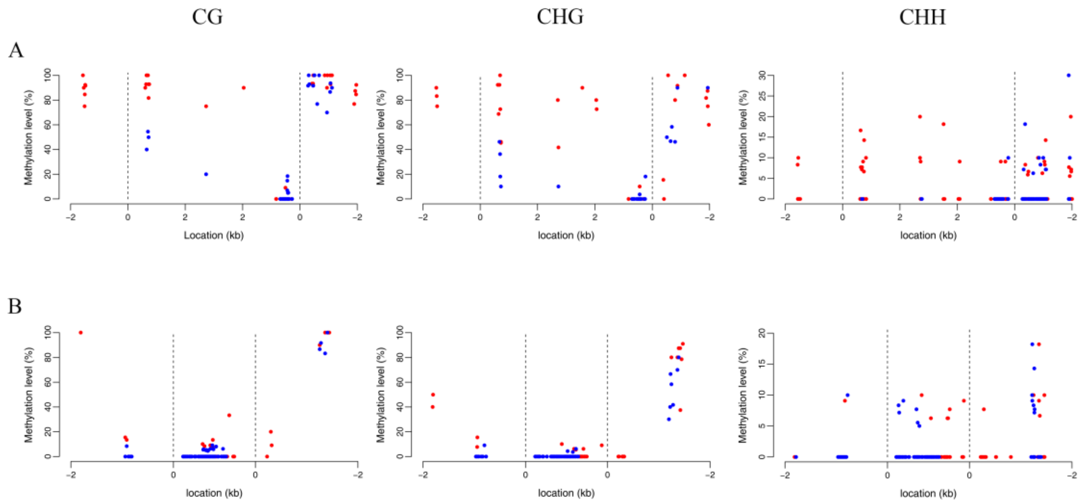

Figure K. DNA methylation patterns of *Fie1* and *floury-1*.

Table A. Statistics of DNA methylation in embryo and endosperm.

	Em	En
Sequences analysed in total	433715164	456749505
Number of alignments with a unique best hit from the different alignments	165285422	191527272
Mapping efficiency	38.11%	41.93%
Sequences with no alignments under any condition	75173762	40418137
Sequences did not map uniquely	193255980	224804096
Total number of C's analysed	2936910521	3523921294
Total methylated C's in CG context	489026492	627830436
Total methylated C's in CHG context	340570370	381748585
Total methylated C's in CHH context	44995351	37574000
Total C to T conversions in CG context	120261124	172940175
Total C to T conversions in CHG context	193182776	281062348
Total C to T conversions in CHH context	1748874408	2022765750
C methylated in CG context	80.26%	78.40%
C methylated in CHG context	63.81%	57.60%
C methylated in CHH context	2.51%	1.82%

Table B. Methylation fraction distribution in embryo and endosperm.

		Em			En		
		CG	CHG	CHH	CG	CHG	CHH
0%	Antisense	4293503	5087086	86323407	4504135	5117335	90991236
	Sense	4289768	5084141	86291308	4500938	5117235	90964097
	Total	8583271	10171227	172614715	9005073	10234570	181955333
	%	13.04%	17.32%	86.61%	12.12%	16.08%	88.73%
1%-20%	Antisense	389175	886309	9765575	444508	1491972	9289758
	Sense	389150	883665	9756451	445078	1491070	9280830
	Total	778325	1769974	19522026	889586	2983042	18570588
	%	1.18%	3.01%	9.79%	1.20%	4.69%	9.06%
21%-40%	Antisense	170451	1645333	3008691	322707	3046446	1916164
	Sense	170198	1644564	3002570	322269	3045821	1915110
	Total	340649	3289897	6011261	644976	6092267	3831274
	%	0.52%	5.60%	3.02%	0.87%	9.57%	1.87%
41%-60%	Antisense	500437	3111204	456696	894823	4937833	266680
	Sense	500761	3113820	455544	895674	4937415	267486
	Total	1001198	6225024	912240	1790497	9875248	534166
	%	1.52%	10.60%	0.46%	2.41%	15.52%	0.26%
61%-80%	Antisense	3399290	5921893	96065	4668486	7556542	69125
	Sense	3396107	5916106	95576	4663807	7551327	68950
	Total	6795397	11837999	191641	9332293	15107869	138075
	%	10.32%	20.16%	0.10%	12.56%	23.74%	0.07%
81%-100%	Antisense	24178240	12708026	27305	26334632	9672563	23903
	Sense	24168352	12706728	27553	26307786	9668368	23547
	Total	48346592	25414754	54858	52642418	19340931	47450
	%	73.42%	43.29%	0.03%	70.85%	30.39%	0.02%
Total	Antisense	32931096	29359851	99677739	37169291	31822691	102556866
	Sense	32914336	29349024	99629002	37135552	31811236	102520020
	Total	65845432	58708875	199306741	74304843	63633927	205076886
Total C detected	Antisense	161968686			171548848		
	Sense	161892362			171466808		
	total	323861048			343015656		
Total C analysised		2,936,910,521			3,523,921,294		
Coverage depth		9.07			10.27		
Total C in maize genome	Antisense	481,515,233					
	Sense	481,097,011					
% coverage	Antisense	33.64%			35.63%		
	Sense	33.65%			35.64%		

Table C. DMR distribution in Embryo and Endosperm.

Annotation		CHG				CHH				CG			
		OB		OT		OB		OT		OB		OT	
Class	type	+	-	+	-	+	-	+	-	+	-	+	-
Type I Transposon/LTR	Gypsy	129425	35847	130138	36030	71452	44096	72382	44105	59161	30510	59201	30571
	Unknown	38770	11734	38131	11280	23818	14845	23879	14609	18090	9740	17848	9379
	Copia	51710	11533	51205	11487	38405	21609	37772	21258	22910	11270	22165	11217
Type II Transposon/TIR	Tc1	111	27	84	27	163	84	165	98	101	41	115	38
	CACTA	10578	3298	10505	3288	7329	4308	7289	4262	5021	3247	5244	3221
	Mutator	4361	1318	4297	1277	3189	1809	3205	1779	2287	994	2240	958
	hAT	3007	1106	3197	1057	2712	1462	2799	1502	1594	833	1645	754
	Pif	1386	452	1387	503	1236	753	1268	854	814	378	851	316
Type I Transposon/LINE	Unknown	1181	486	1156	489	983	619	977	638	703	344	723	334
	L1	147	62	114	36	120	69	94	56	77	48	68	20
Transposable Element	upstream	5040	1348	4658	1331	3374	1926	3243	1872	2554	1140	2356	1050
	gene body	3601	808	3415	849	2194	1247	2153	1150	1878	660	1739	690
	downstream	4356	1242	4317	1163	3118	1694	2998	1711	2202	1059	2175	1009
Pseudogene	upstream	2774	787	2859	744	2314	1226	2297	1266	1634	614	1591	588
	gene body	628	187	661	168	537	309	517	277	365	135	378	120
	downstream	2745	746	2752	757	2325	1211	2330	1258	1642	599	1664	613
Protein-coding gene	upstream	11229	3776	11617	3872	10091	5849	10511	6009	6537	2755	6648	2693
	gene body	12397	4217	12611	4510	16165	9804	16706	10127	11864	3833	11730	3766
	downstream	9621	3278	9636	3408	9571	5365	9564	5502	6038	2334	5980	2391

Table D. Statistics of DMR between embryo and endosperm.

			Site number	Base number
CG	Antisense	+	285017	16867200
		-	136120	7474400
	Total		421137	24341600
	% of +		67.68%	69.29%
	Sense	+	281796	16700200
		-	133694	7341750
	Total		415490	24041950
	% of +		67.82%	69.46%
CHG	Antisense	+	580449	38747900
		-	157885	8654600
	Total		738334	47402500
	% of +		78.62%	81.74%
	Sense	+	578949	38707150
		-	157313	8627900
	Total		736262	47335050
	% of +		78.63%	81.77%
CHH	Antisense	+	364486	20285400
		-	213228	11373300
	Total		577714	31658700
	% of +		63.09%	64.08%
	Sense	+	364239	20313750
		-	212770	11350950
	Total		577009	31664700
	% of +		63.13%	64.15%

"+" represents that the DMR is highly methylated in embryo, whereas "-" means the DMR is highly methylated in endosperm.

Table E. Gene expression in embryo and endosperm.

Gene name	Transcript ID	RPKM	
		Em	En
Fie1	GRMZM2G118205_T01	0.08	27.24
Floury1	GRMZM2G094532_T01	0.74	20.47
